# Introducing the glycyrrhizic acid and glabridin rich genotypes from the cultivated Iranian licorice (*Glycyrrhiza glabra* L.) populations to exploit in production systems

**DOI:** 10.1038/s41598-024-61711-1

**Published:** 2024-05-14

**Authors:** Hassan Esmaeili, Mohammad Hossein Mirjalili, Akbar Karami, Samad Nejad Ebrahimi

**Affiliations:** 1 Department of Agriculture, Medicinal Plants and Drugs Research Institute, Shahid Beheshti University, Tehran, 1983969411 Iran; 2 Department of Horticultural Science, School of Agriculture, Shiraz University, Shiraz, Iran; 3 Department of Phytochemistry, Medicinal Plants and Drugs Research Institute, Shahid Beheshti University, Tehran, 1983969411 Iran

**Keywords:** Fabaceae, Flavonoid, Liquorice, Phytochemical analysis, Specialized metabolites, Ecology, Evolution

## Abstract

Currently, the stable, uniform, and highly efficient production of raw materials for pharmaceutical companies has received special attention. To meet these criteria and reduce harvesting pressure on the natural habitats of licorice (*Glycyrrhiza glabra* L.), cultivation of this valuable plant is inevitable. In the present study, to introduce the glycyrrhizic acid (GA)- and glabridin-rich genotypes from cultivated Iranian licorice, forty genotypes from eight high-potential wild populations were cultivated and evaluated under the same environmental conditions. The GA content varied from 5.00 ± 0.04 mg/g DW (TF2 genotype) to 23.13 ± 0.02 mg/g DW (I5 genotype). The highest and lowest glabridin content were found in the K2 (0.72 ± 0.021 mg/g DW) and M5 (0.02 ± 0.002 mg/g DW) genotypes, respectively. The rutin content in the leaves of the studied genotypes varied from 1.27 ± 0.02 mg/g DW in E4 to 3.24 ± 0.02 mg/g DW in BO5 genotypes. The genotypes from the Ilam population were characterized by higher vegetative growth and yield traits in the aerial parts and roots. The average root dry yield was 2.44 tons per hectare (t/ha) among the studied genotypes and a genotype from Ilam (I5) yielded the maximum value (3.08 ± 0.034 t/ha). The highest coefficient of variation among the genotypes was observed for leaf width (CV = 34.9%). The GA and glabridin-rich genotypes introduced in this study can be used in the future breeding programs to release new bred licorice cultivars.

## Introduction

Licorice (*Glycyrrhiza glabra* L.) is an industrial herb with underground creeping stems that grows in wide areas of the globe from southern Europe to eastern Asia^1^. The plant underground extract is widely utilized in the food, tobacco, pharmaceutical, and cosmetic industries. Consumption in the preparation of various drinks, sweets, and chewing gum are examples of the licorice applications in the food industry^2^. Licorice possesses numerous medicinal effects such as anti-ulcer, hepatoprotective, anti-oxidant, anti-microbial, anti-cancer, anti-inflammatory, memory enhancing, hypocholesterolemic, and anti-depressant effects which have also been reviewed^3^.

**Figure 1:**
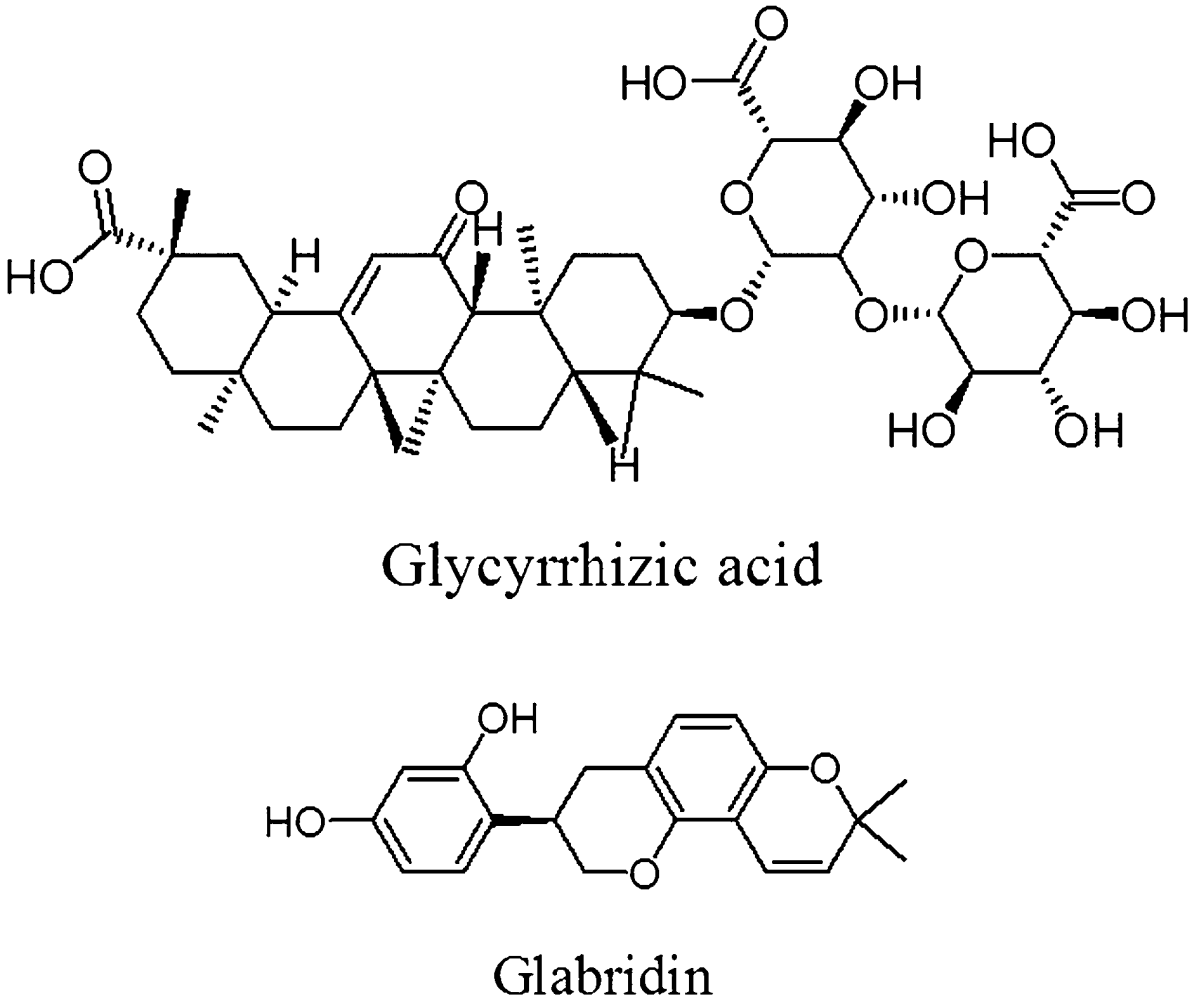
The chemical structure of two valuable phytochemical constituents found in the underground of licorice (*Glycyrrhiza glabra* L*.*).

The plant embraces well-known bioactive constituents including triterpenoids, flavonoids, phenolics, and coumarins^4^. Glycyrrhizic acid (GA) and glabridin (Fig. 1) are two prominent ingredients in the underground parts of the plant with extensive applications. Glycyrrhizic acid (glycyrrhizin), a glucosiduronide derivative of 3beta-hydroxy-11-oxoolean-12-en-30-oic acid (C_42_H_62_O_16_) with an amphiphilic structure, is extensively used as a sweetener in foods, sweets, and candies. The underground parts of the plant used in different preparations and products are evaluated based on the GA content as an important index recorded in the most important international pharmacopeia. Anticancer, antiviral, anti-inflammatory, and hepatoprotective activities of GA have been previously reported. The potential healing effects of GA on liver and skin diseases have also been demonstrated^5–8^.

Glabridin, a prenylated isoflavan (C_20_H_20_O_4_), is another valuable phytochemical compound isolated from the plant’s underground parts which have received considerable attention in recent years. Glabridin is the most important flavonoid in licorice and is considered a phytoestrogen compound. Antioxidant, anti-inflammatory, neuroprotective, anti-osteoporosis, and skin-whitening effects of this flavonoid have also been reported^9–11^. This compound is also useful for the treatment of central nervous system and cardiovascular complaints^12^. Glabridin also causes pigment-lightening by inhibiting tyrosinase activity^13^. Until 2013, more than 160 inventions related to various aspects of extraction, purification, formulation, and various products containing glabridin have been registered^14^.

The interaction between the environment and genotype determines the quantity and quality of the plants’ specialized metabolites (SMs)^15^. Various accessions of medicinal and aromatic plants (MAPs) that have grown in diverse ecological zones form different ecotypes along with ecotype-specific biological characteristics. When a plant population is initially exposed to a particular environmental condition, its physiological behavior changes to endure the new environment^16^. Gradually, owing to the force of evolution, a population may acquire distinct physiological, chemical, botanical, and genetic characteristics^17^. The stable, uniform, and highly efficient production of raw materials for pharmaceutical companies has currently, received special attention^18^. However, the innumerable and uncontrolled harvesting of plants from nature has exposed them to extinction.

To meet these criteria, domestication and introduction of wild MAPs into the cultivation systems are critically needed. It should be considered that the first and most important step in the domestication strategy is carefully monitoring the phytochemical, morphological, and genetic aspects of wild MAPs accessions^19^. The next step is to select the most desirable wild accessions followed by cultivation to evaluate them under new and uniform environmental conditions. The breeder pursues its intended breeding goals during the domestication process and exploits the existing diversity among the accessions. About licorice, According to the reports of the Ministry of Agriculture of Iran, there are about 2800 hectares of irrigated and 240 hectares of rainfed licorice cultivation limited to a few provinces. This cultivated area with the production of 12,611.7 tons cannot meet the needs of the extraction facilities. On the other hand, in most parts of Iran, strict laws have been established by the government to harvest the plant from nature. Therefore, in order to supply raw materials for factories, extensive cultivation of the plant and planning for breeding programs to increase the quality of effective compounds should be on the agenda in Iran.

The content and composition of active ingredients, growth form and vigor, the ratio of the medicinal organ to the whole plant, and resistance to biotic and abiotic stresses are the most emphasized goals in the selection of high-quality MAPs accessions during domestication and breeding efforts^20^. Approximately, 75% of MAPs are currently harvested from their natural habitats and therefore the heterogeneity of the plants bioactive compounds, a result of environmental effects, and ecosystem degradation are the problems that human beings face^21^.

Three types of licorice, including *Glycyrrhiza uralensis*, *Glycyrrhiza inflata* and *Glycyrrhiza glabra* are known as medicinal licorice, of which *G. uralensis* accounts for 90% of production^22^. Due to indiscriminate harvesting, successive droughts, human activities, and the consequent reduced reproduction as well, the plant ncritically decreased while the demand pressure for the plant materials is still existing. For these reasons, parts of northwest China have been dedicated to the cultivation of this plant and known as alternative source of wild licorice^23^. Cultivation of licorice has grown since 2007 with a 30% increase in its price in China^24^. Drastic reduction in the wild resources of the licorice has happened in China, Middle East and Eurasia countries^25^. The licorice cultivation in the Aral Sea regions in Uzbekistan is also a successful example of the cultivation of this plant in harsh environmental conditions^26^.

During the previous researches, wild populations of licorice were collected across Iran and subjected to genetic, phytochemical, and phenotypic evaluations^27,28^. The identification of desirable populations under cultivation conditions was then targeted. After identifying the desired populations, efforts were focused on identifying and introducing superior genotypes for cultivation and commercial exploitation on a large scale not only for Iran but also for countries prone to growing licorice in the Eurasian and Caucasus regions.

In addition to GA, which has been discussed in extraction, purification, and pharmacological aspects, in recent years, glabridin has gained special importance. Given the importance of GA and glabridin as well as the increasing demand for their utilization in industry, the present study aimed to introduce GA- and glabridin-rich genotypes from cultivated Iranian *G. glabra* populations. This study not only introduced the superior genotypes in terms of phytochemicals but also focused on the comparison of these medicinal compounds in cultivated and wild genotypes. These introduced genotypes can be used in the future breeding programs to release new bred licorice cultivars for commercial cultivation.

## Materials and methods

### Chemical and reagents

Rutin, gallic acid, 2, 2-diphenyl-1-picryl-hydrazyl (DPPH), GA, glabridin, liquiritin, and liquiritigenin standards were purchased from Phytopurify Company, China. HPLC-grade acetonitrile and methanol were prepared by Biochem Chemopharma Company.

### Field trial and cultivation conditions

The identical rhizomes (with a diameter of 1.5 cm and a length of 20–25 cm) of forty genotypes from eight wild licorice populations that were recognized as GA and glabridin-rich populations in their habitat^27^ were planted in pots in the greenhouse. The permission for harvesting the wild licorice genotypes was obtained from the Iranian Organization of Forests and Rangeland. The sampling was performed according to national and International Standards for Sustainable Wild Collection of Medicinal and Aromatic Plants (ISSC-MAP) (Version 1.0), and the Medicinal Plant Specialist Group Species Survival Commission of IUCN (The World Conservation Union). The plant populations were botanically identified by Prof. Sonboli and voucher specimens have been deposited at the Medicinal Plants and Drugs Research Institute Herbarium of Shahid Beheshti University. The voucher numbers (from MPH-3216 to MPH-3223) for plant populations are presented in Table 1. The all forty raised genotypes (five individuals from each population) were then transferred and cultivated (30 × 70 cm) in a randomized block design (RCBD) experiment at the research farm of the Medicinal Plants and Drugs Research Institute (MPDRI), Shahid Beheshti University, Evin, Tehran, Iran (35°48′285″ N, 51°23′494″ E and altitude 1785 m) in March 2019 with three replications. The climatic conditions of the natural habitat of *G. glabra* have been shown in Table 1. The environmental and edaphic specifications of the research farm are also given in Table 2.

**Table 1:** Climatic conditions of natural habitats of the studied Iranian *Glycyrrhiza glabra* genotypes.

Genotype code	Population	Voucher Number	Altitude (m)	Average annual temperature (°C)	Average annual precipitation (mm)
K1–K5	Kashmar	MPH-3222	1632	17.8	197.2
TK1–TK5	Takestan	MPH-3223	1265	16.1	302
TF1–TF5	Taft	MPH-3219	2286	19.2	59.2
B1–B5	Bajgah	MPH-3217	1798	16.4	325.3
E1–E5	Eghlid	MPH-3216	2319	12.9	315.5
M1–M5	Marvest	MPH-3218	1542	17.8	64.6
I1–I5	Ilam	MPH-3220	1032	16.9	583.7
BO1–BO5	Bojnourd	MPH-3221	970	13.3	267.8

**Table 2:** Environmental and edaphic characteristics of the cultivation site.

Meteorological and climatic conditions	Latitude (N)	35° 48′ 20.6″
	Longitude (E)	51° 23′ 35.3″
	Altitude (m)	1190
	AT (°C)	17.3
	MAT (°C)	13.3
	MXAT (°C)	22.9
	AP (mm)	395.5
Edaphological characteristics	N_total_ (%)	0.26
	P_ava_ (ppm)	66.5
	K_ava_ (ppm)	350
	pH	7.8
	EC (ds/m)	0.71
	OC (%)	2.04
	% Sand	49
	% Silt	32
	% Clay	19
	Texture	Loamy

*AT* mean annual temperature, *MAT* average minimum annual temperature, *MXAT* average maximum annual temperature, *AP* mean annual precipitation.

### Morphological analysis

The aerial parts of the genotypes were sampled in the early flowering stage and evaluated for morphological traits such as aerial part fresh weight, plant height, plant and stem diameter, number of branches and nods, and leaf length and width. The collected samples were dried in the shade at ambient temperature and the dry weight was recorded. The underground parts of the plants were harvested in autumn simultaneously with the onset of plant dormancy and the root fresh and dry weights were measured. To determine the above-mentioned traits, a sensitive digital scale and caliper were used.

### Preparation of underground and aerial part extracts

Dried pulverized underground part of the plant samples (500 mg) were macerated and extracted using 10 ml Ethanol: water (70:30) and sonication. Ethanol: water (70:30) was used to extract licorice metabolites according to an initial test to identify the appropriate solvent for the simultaneous extraction of glabridin and GA as described previously^27^. Extraction of aerial parts for rutin measurement was performed using methanol and then filtered and injected into the HPLC.

### Quantitative analysis by HPLC-PDA

A Waters liquid chromatography device coupled with a Photodiode Array Detector (PDA) and C_18_ column (Knauer, 25 cm × 4.6 mm Eurospher 100-5) was used for phytochemical quantification of the plant materials (the aerial and underground parts) according to Esmaeili et al.^27^.

### Total phenol and total flavonoid measurement

Total phenol content (TPC) was calculated by the Folin–Ciocalteu procedure using a spectrophotometer at a wavelength of 760 nm with three replications as previously described by Lister and Wilson^29^. Gallic acid was applied to plot a calibration curve. TPC was calculated and reported as milligram gallic acid equivalent per gram of plant dry weight (mg GAE/g dry weight).

The total flavonoid content (TFC) was also determined using the same spectrophotometer device mentioned above according to the method of Zhishen et al.^30^ at a wavelength of 510 nm. TFC was reported as milligram rutin (as standard) equivalent per gram of plant dry weight (mg rutin/g dry weight).

### Assay of antioxidant activity

The antioxidant potential of the extracts was evaluated by the 2, 2-diphenyl-1-picrylhydrazyl (DPPH) method at 517 nm according to the following equation: inhibition % = (Ac − As)/Ac*100 where, As is the sample absorbance and Ac is the control absorbance which as previously described by Blois^31^. The FRAP (Ferric Reducing Antioxidant Power) method was also applied for total antioxidant activity of the genotypes roots. Briefly, a mixture of acetate buffer, TPTZ solution, and iron chloride solution was prepared. 200 µl of this solution was poured to the plate wells and then 20 µl of the root extract was added to each well. The plate was incubated at 37 °C on a shaker in dark condition for 30 min. The absorbance was recorded at 593 nm.

### Statistical analysis

The coefficient of variation (CV%) was calculated to evaluate the diversity of morphological traits among genotypes. The analysis of variance (ANOVA) and the mean comparison of the measured traits using Duncan’s multiple-range test were performed in SPSS at a confidence level of 95% (*p* ≤ 0.05). Cluster heatmap analysis, correlation analysis, and Principal component analysis (PCA) were performed using R software.

## Results

### Yield and morphological traits

The result of analysis of variance (ANOVA) for yield and morphological traits was presented in Table S1. In the present study, the morphological traits among the genotypes revealed high variation. The dissection of morphological traits of cultivated genotypes revealed that the maximum values for fresh and dry weight of the plant aerial part, and root fresh weight belonged to genotype I3. The plant height among the studied genotypes was also varied from 15.63 ± 0.51 cm in TF2 genotype to 82.13 ± 0.2 cm in I1 genotype. The highest and lowest numbers of branches were recorded for genotypes M5 and I1, respectively. The largest leaves along with maximum leaf length (36.23 ± 0.81 mm) and width (12.90 ± 0.2 mm) were observed in the genotype of the Takestan population (TK5). All five genotypes from the Taft population were distinguished from other genotypes by weak vegetative growth, and the lowest fresh and dry weights in the aerial parts and roots (Table 3).

**Table 3:** Some studied morphological and yield traits among cultivated *Glycyrrhiza glabra* genotypes.

Genotype code	Aerial part fresh weight (g)	Aerial part dry weight (g)	Plant height (cm)	Plant diameter (cm)	Stem diameter (mm)	No. of branches	No. of node	Leaf length (mm)	Leaf width (mm)	Root fresh weight (g)	Root dry weight (g)	Root yield (t/ha)
K1	64.63 ± 1.5	34.23 ± 1.11	67.70 ± 0.9	26.77 ± 0.95	6.10 ± 0.60	9.67 ± 0.58	8.33 ± 0.58	30.57 ± 0.80	11.20 ± 0.3	77.90 ± 0.96	51.37 ± 0.15	2.45 ± 0.007
K2	64.70 ± 1.2	35.63 ± 0.91	68.70 ± 1.7	27.33 ± 0.86	5.03 ± 0.32	10.67 ± 0.58	10.67 ± 1.2	31.40 ± 0.70	10.87 ± 0.4	81.33 ± 0.68	53.10 ± 0.40	2.52 ± 0.010
K3	66.47 ± 1.0	33.73 ± 0.91	66.40 ± 1.7	28.10 ± 1.00	5.80 ± 0.60	9.33 ± 1.53	11.00 ± 1.0	28.23 ± 0.49	10.17 ± 0.3	77.73 ± 0.65	51.97 ± 0.51	2.47 ± 0.024
K4	63.37 ± 1.6	31.63 ± 0.75	62.00 ± 7.0	24.77 ± 1.51	6.20 ± 0.46	10.67 ± 1.15	7.33 ± 0.6	29.93 ± 0.47	11.07 ± 0.3	78.70 ± 1.04	52.70 ± 0.40	2.50 ± 0.010
K5	67.87 ± 1.5	34.10 ± 0.62	69.10 ± 0.6	25.20 ± 0.90	6.23 ± 0.42	11.33 ± 0.58	9.33 ± 0.6	30.47 ± 0.85	10.93 ± 0.3	80.47 ± 0.84	52.53 ± 0.50	2.51 ± 0.013
TK1	76.23 ± 1.0	41.40 ± 0.62	70.53 ± 1.4	24.83 ± 0.31	5.33 ± 0.15	9.33 ± 0.58	8.00 ± 1.0	34.53 ± 0.65	12.53 ± 0.2	77.73 ± 0.47	48.20 ± 0.56	2.28 ± 0.011
TK2	76.20 ± 0.9	36.67 ± 0.80	67.83 ± 1.5	23.63 ± 0.51	6.50 ± 0.40	9.33 ± 1.53	7.33 ± 0.6	32.93 ± 0.50	12.63 ± 0.2	79.63 ± 0.47	48.77 ± 0.81	2.34 ± 0.019
TK3	72.50 ± 0.9	35.37 ± 1.31	66.57 ± 1.4	22.03 ± 0.42	5.17 ± 0.31	7.33 ± 0.58	7.67 ± 1.2	35.53 ± 1.12	12.23 ± 0.4	76.53 ± 0.45	47.70 ± 0.26	2.27 ± 0.004
TK4	73.40 ± 1.2	42.60 ± 0.50	71.07 ± 0.7	23.33 ± 0.35	6.50 ± 0.36	9.67 ± 1.53	9.67 ± 1.2	34.37 ± 1.22	12.23 ± 0.4	78.40 ± 0.44	47.53 ± 0.21	2.26 ± 0.007
TK5	73.30 ± 1.4	43.23 ± 1.03	71.70 ± 1.2	25.20 ± 0.46	4.87 ± 0.65	9.33 ± 0.58	7.33 ± 0.6	36.23 ± 0.81	12.90 ± 0.2	79.10 ± 0.50	48.63 ± 0.45	2.31 ± 0.022
TF1	48.67 ± 0.9	27.07 ± 1.17	52.83 ± 1.3	18.53 ± 0.67	3.63 ± 0.15	9.00 ± 1.00	8.33 ± 0.6	20.27 ± 1.06	5.57 ± 0.1	59.63 ± 0.81	39.60 ± 0.46	1.88 ± 0.008
TF2	46.67 ± 1.1	24.97 ± 1.69	47.87 ± 0.3	15.63 ± 0.51	3.60 ± 0.10	9.33 ± 0.58	8.67 ± 1.5	20.97 ± 0.67	5.77 ± 0.2	59.03 ± 0.31	38.93 ± 0.25	1.86 ± 0.009
TF3	50.40 ± 1.2	26.43 ± 0.68	52.07 ± 2.4	18.47 ± 0.55	3.83 ± 0.06	6.67 ± 0.58	7.33 ± 0.6	19.60 ± 0.50	5.37 ± 0.2	58.67 ± 0.55	38.77 ± 0.25	1.84 ± 0.012
TF4	47.37 ± 1.1	23.37 ± 0.55	50.00 ± 1.4	16.83 ± 0.49	3.57 ± 0.06	7.33 ± 0.58	8.33 ± 0.6	19.30 ± 0.72	5.53 ± 0.1	60.33 ± 1.16	40.10 ± 0.40	1.91 ± 0.015
TF5	49.50 ± 1.6	25.80 ± 1.05	51.80 ± 2.1	18.03 ± 1.08	3.87 ± 0.06	7.67 ± 1.15	7.33 ± 1.1	21.37 ± 0.47	5.27 ± 0.1	58.53 ± 0.55	39.33 ± 0.42	1.86 ± 0.007
B1	76.63 ± 1.3	44.87 ± 1.33	64.60 ± 1.7	22.90 ± 0.20	5.40 ± 0.20	10.33 ± 0.58	9.00 ± 1.0	28.27 ± 0.45	5.50 ± 0.1	96.73 ± 0.40	57.33 ± 0.78	2.72 ± 0.015
B2	73.90 ± 2.7	39.63 ± 1.10	64.73 ± 2.0	20.60 ± 1.01	5.73 ± 0.15	9.33 ± 0.58	8.33 ± 1.1	27.83 ± 0.87	6.00 ± 0.1	96.07 ± 0.40	56.77 ± 0.61	2.69 ± 0.020
B3	74.33 ± 0.9	38.70 ± 1.20	64.40 ± 1.0	22.30 ± 1.06	5.27 ± 0.15	10.67 ± 0.58	8.00 ± 1.0	28.97 ± 0.51	5.30 ± 0.1	95.40 ± 0.36	56.43 ± 0.35	2.68 ± 0.007
B4	77.60 ± 1.0	44.03 ± 0.76	68.20 ± 0.3	22.67 ± 1.90	5.67 ± 0.15	9.00 ± 1.00	10.00 ± 1.0	27.10 ± 0.50	6.07 ± 0.2	97.17 ± 0.47	58.57 ± 0.42	2.78 ± 0.014
B5	75.57 ± 1.2	38.93 ± 1.12	64.23 ± 2.3	21.07 ± 1.48	5.47 ± 0.21	9.00 ± 2.00	9.67 ± 1.6	28.10 ± 0.30	5.53 ± 0.1	96.00 ± 0.44	57.17 ± 0.45	2.73 ± 0.012
E1	65.33 ± 1.3	36.37 ± 1.36	65.57 ± 1.1	26.80 ± 1.21	4.63 ± 0.15	12.00 ± 1.00	14.00 ± 1.0	23.00 ± 0.56	7.67 ± 0.1	79.13 ± 0.35	47.33 ± 0.32	2.25 ± 0.004
E2	62.03 ± 0.5	33.57 ± 1.03	62.33 ± 1.0	26.40 ± 2.12	5.17 ± 0.12	11.67 ± 0.58	11.00 ± 1.0	22.87 ± 0.57	7.33 ± 0.2	78.33 ± 0.21	47.10 ± 0.40	2.24 ± 0.010
E3	62.00 ± 1.5	33.60 ± 1.35	61.30 ± 1.2	28.63 ± 0.75	5.13 ± 0.06	10.67 ± 1.15	12.67 ± 0.6	22.13 ± 0.32	7.90 ± 0.2	80.53 ± 0.35	48.53 ± 0.40	2.31 ± 0.019
E4	67.80 ± 1.4	38.40 ± 0.53	65.33 ± 1.1	26.73 ± 1.11	5.10 ± 0.20	12.00 ± 1.00	13.00 ± 1.0	23.43 ± 0.35	7.83 ± 0.3	80.90 ± 0.89	48.57 ± 0.67	2.32 ± 0.012
E5	64.83 ± 1.4	35.17 ± 0.70	62.60 ± 0.6	26.93 ± 1.72	4.47 ± 0.06	10.00 ± 1.00	12.67 ± 0.6	23.00 ± 0.30	7.50 ± 0.2	80.03 ± 0.60	47.67 ± 0.47	2.27 ± 0.020
M1	71.43 ± 1.1	39.23 ± 0.61	56.83 ± 0.5	32.13 ± 1.24	4.47 ± 0.15	15.67 ± 1.15	14.67 ± 0.5	29.57 ± 0.50	5.77 ± 0.15	89.17 ± 0.83	54.97 ± 0.76	2.61 ± 0.033
M2	68.43 ± 1.0	37.40 ± 1.21	54.00 ± 0.5	27.67 ± 0.80	4.27 ± 0.06	15.67 ± 0.58	13.67 ± 0.6	31.03 ± 0.57	5.53 ± 0.15	90.77 ± 0.25	56.17 ± 0.21	2.67 ± 0.010
M3	70.13 ± 0.7	39.93 ± 1.00	55.53 ± 1.2	31.90 ± 1.06	4.50 ± 0.10	17.33 ± 0.58	13.67 ± 1.5	28.57 ± 0.47	5.57 ± 0.15	89.33 ± 0.65	55.17 ± 0.50	2.63 ± 0.024
M4	68.63 ± 0.4	36.47 ± 1.12	54.53 ± 1.1	29.33 ± 1.64	4.43 ± 0.12	15.00 ± 1.00	13.67 ± 0.5	30.27 ± 0.85	6.17 ± 0.15	90.03 ± 0.99	54.63 ± 0.47	2.60 ± 0.021
M5	72.37 ± 1.0	40.97 ± 1.16	58.13 ± 0.8	29.97 ± 1.86	4.33 ± 0.06	17.67 ± 1.15	14.33 ± 0.5	29.13 ± 0.61	5.73 ± 0.15	91.10 ± 0.40	55.27 ± 0.15	2.63 ± 0.007
I1	85.37 ± 0.9	46.60 ± 1.15	82.13 ± 0.2	26.33 ± 1.12	6.50 ± 0.10	5.00 ± 1.00	8.00 ± 1.0	30.10 ± 0.30	8.13 ± 0.21	105.07 ± 0.31	64.40 ± 0.36	3.06 ± 0.012
I2	83.50 ± 0.8	44.53 ± 1.21	78.73 ± 1.1	23.67 ± 0.93	6.67 ± 0.15	7.67 ± 0.58	7.33 ± 0.6	32.10 ± 0.40	8.17 ± 0.15	104.73 ± 0.21	63.83 ± 0.15	3.03 ± 0.004
I3	85.97 ± 1.2	46.87 ± 0.65	80.97 ± 1.1	27.90 ± 1.04	6.40 ± 0.10	6.67 ± 0.58	7.00 ± 1.0	31.80 ± 0.66	7.80 ± 0.10	105.90 ± 0.30	62.47 ± 0.21	2.97 ± 0.010
I4	84.27 ± 1.2	44.77 ± 0.38	76.10 ± 1.0	25.53 ± 0.95	6.77 ± 0.12	7.67 ± 0.58	8.67 ± 0.6	31.77 ± 0.60	7.90 ± 0.20	103.90 ± 0.26	64.60 ± 0.30	3.07 ± 0.007
I5	85.47 ± 1.0	45.37 ± 0.74	78.17 ± 2.5	24.83 ± 1.38	6.63 ± 0.15	8.00 ± 1.00	7.00 ± 1.0	32.57 ± 0.32	8.27 ± 0.15	103.73 ± 0.25	64.77 ± 0.76	3.08 ± 0.034
BO1	62.53 ± 1.0	34.00 ± 0.75	53.07 ± 1.1	19.10 ± 0.70	4.23 ± 0.06	11.00 ± 1.00	9.00 ± 1.0	17.90 ± 0.20	4.77 ± 0.15	79.13 ± 0.32	47.50 ± 0.46	2.25 ± 0.015
BO2	60.13 ± 0.9	33.50 ± 0.96	51.67 ± 1.3	22.27 ± 1.11	4.27 ± 0.21	10.00 ± 1.00	10.33 ± 0.6	18.93 ± 0.42	4.57 ± 0.15	78.30 ± 0.44	46.53 ± 0.21	2.21 ± 0.007
BO3	64.33 ± 0.7	35.97 ± 0.40	53.60 ± 0.1	18.60 ± 1.40	4.00 ± 0.26	11.67 ± 0.58	8.00 ± 1.0	19.67 ± 0.25	4.93 ± 0.15	78.23 ± 0.32	45.63 ± 0.47	2.17 ± 0.021
BO4	62.83 ± 1.0	34.17 ± 0.86	52.17 ± 0.5	21.40 ± 1.15	4.33 ± 0.15	9.00 ± 1.00	9.33 ± 0.5	18.37 ± 0.40	4.40 ± 0.10	79.53 ± 0.35	46.57 ± 0.42	2.21 ± 0.019
BO5	64.17 ± 1.1	34.37 ± 1.17	53.00 ± 1.1	21.63 ± 1.76	4.17 ± 0.25	10.00 ± 1.00	10.00 ± 1.0	18.90 ± 0.44	5.07 ± 0.15	79.13 ± 0.35	46.13 ± 0.40	2.20 ± 0.012
Mean	68.27	36.84	62.95	24.15	5.11	10.23	9.74	27.03	7.59	83.30	51.29	2.44
SD	10.34	6.02	9.17	4.04	0.98	2.80	2.37	5.49	2.65	13.06	7.12	0.34
CV %	15.14	16.34	14.56	16.73	19.16	27.39	24.37	20.30	34.97	15.67	13.89	13.89

**Figure 2:**
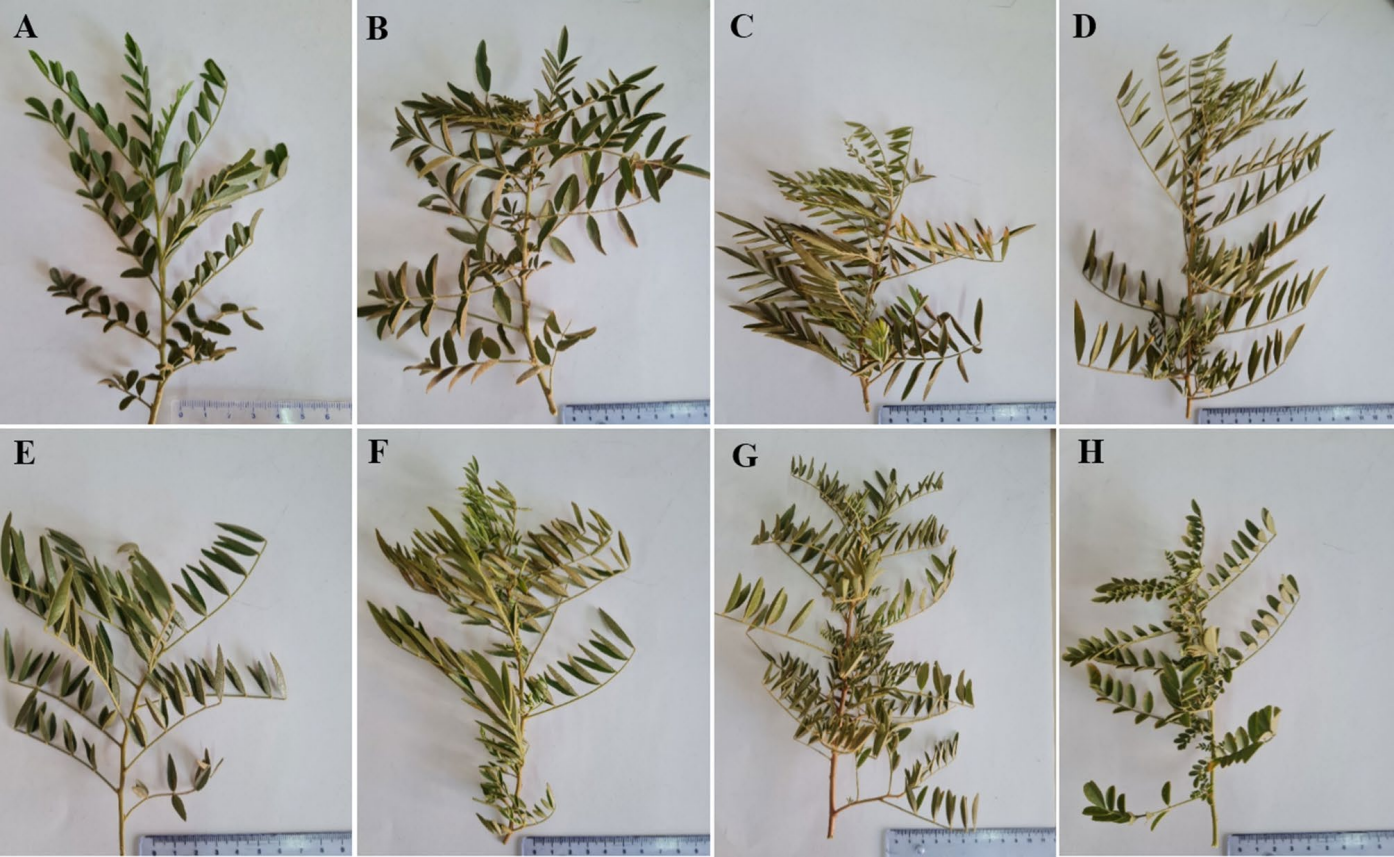
The leaf variation of cultivated licorice (*Glycyrrhiza glabra* L*.*) populations. (**A**) Kashmar; (**B**) Takestan; (**C**) Taft; (**D**) Bajgah; (**E**) Eghlid; (**F**) Marvest; (**G**) Ilam; (**H**) Bojnourd.

The highest diversity coefficient among the studied genotypes was evident for leaf width (CV = 34.9%). Leaf characteristics did not show much variation within the genotypes of each population, whereas there was significant variation among the genotypes of the different populations. The variation in leaf populations is shown in Fig. 2. The genotypes also showed the least variation in terms of root dry weight and yield (CV = 13.9%). The root dry yield ranged from 1.84 ± 0.012 (t/ha) in the TF3 genotype to 3.08 ± 0.034 (t/ha) in the I5 genotype with an average of 2.44 (t/ha).

### Phytochemical variation

The analysis of variance (ANOVA) for phytochemical traits was presented in Table S2. Phytochemical quantification of the underground part of licorice among the cultivated genotypes revealed that the I5 genotype had the highest GA. The GA content varied from 5.00 ± 0.04 mg/g DW (TF2 genotype) to 23.13 ± 0.02 mg/g DW.

**Figure 3:**
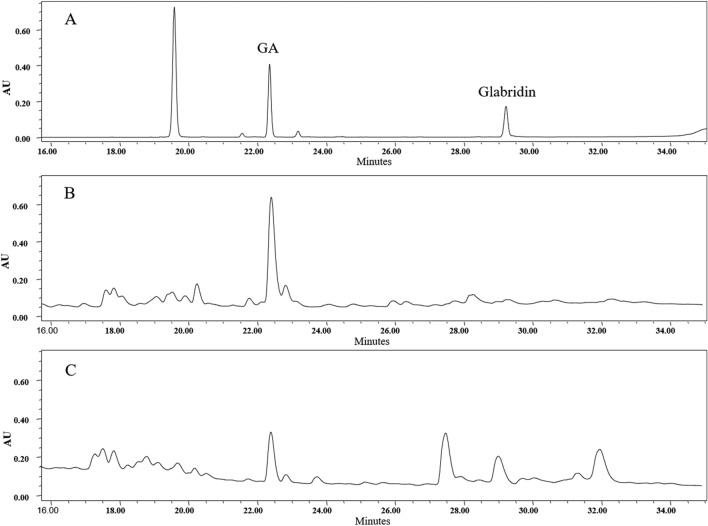
The HPLC-PAD chromatograms of the standards compounds analyzed in the present study (**A**), the ethanolic extract of glycyrrhizic acid-rich genotype (I5) monitored at wavelength 250 nm (**B**), and glabridin-rich genotype (K2) monitored at wavelength 230 nm (**C**).

The glabridin content varied from 0.02 ± 0.002 mg/g DW to 0.72 ± 0.021 mg/g DW among the studied genotypes, wherein the M5 and K2 genotypes had the lowest and highest values, respectively. The liquiritin was detected in B1–B5 genotypes of the Bajgah population (0.16–0.21 mg/g DW) among the studied genotypes. Liquiritigenin content was traced in the cultivated genotypes from Taft, Kashmar, and Takestan populations, while the genotype BO1 from the Bojnourd population contained the highest level (1.76 ± 0.09 mg/g DW) (Table 4). Typical HPLC-PAD chromatograms of the genotypes rich in GA and glabridin are shown in Fig. 3.

**Table 4:** The phytochemical variation in the aerial and underground parts of the cultivated Iranian *Glycyrrhiza glabra* genotypes.

Genotype code	Glycyrrhizic acid (mg/g DW)	Glabridin (mg/g DW)	Liquiritin (mg/g DW)	Liquiritigenin (mg/g DW)	Rutin (mg/g DW)
K1	21.54 ± 0.06^f^	0.68 ± 0.012^bc^	Trace	Trace	2.24 ± 0.03^f^
K2	21.49 ± 0.04^fg^	0.72 ± 0.021^a^	Trace	Trace	2.22 ± 0.03^fg^
K3	21.52 ± 0.03^fg^	0.66 ± 0.015^c–e^	Trace	Trace	2.20 ± 0.02^g^
K4	21.46 ± 0.02^g^	0.67 ± 0.032^cd^	Trace	Trace	2.19 ± 0.01^g^
K5	21.48 ± 0.05^fg^	0.70 ± 0.012^ab^	Trace	Trace	2.15 ± 0.01^h^
TK1	8.50 ± 0.03^k^	0.22 ± 0.026^k–m^	Trace	Trace	1.49 ± 0.05^s^
TK2	8.45 ± 0.02^k^	0.20 ± 0.031^l–o^	Trace	Trace	1.49 ± 0.04^s^
TK3	8.44 ± 0.05^k^	0.26 ± 0.010^j^	Trace	Trace	1.54 ± 0.02^pq^
TK4	8.48 ± 0.05^k^	0.23 ± 0.020^kl^	Trace	Trace	1.52 ± 0.02^qr^
TK5	8.48 ± 0.03^k^	0.21 ± 0.006^k–n^	Trace	Trace	1.49 ± 0.03^rs^
TF1	5.04 ± 0.07^no^	0.23 ± 0.015^k^	Trace	Trace	2.12 ± 0.03^hi^
TF2	5.00 ± 0.04^o^	0.19 ± 0.010^n–p^	Trace	Trace	2.12 ± 0.02^hi^
TF3	5.05 ± 0.03^no^	0.18 ± 0.015^o–q^	Trace	Trace	2.13 ± 0.02^h^
TF4	5.10 ± 0.02^n^	0.21 ± 0.015^k–o^	Trace	Trace	2.07 ± 0.01^ij^
TF5	5.02 ± 0.05^o^	0.16 ± 0.015^p–t^	Trace	Trace	2.09 ± 0.02^ij^
B1	16.77 ± 0.03^h^	0.16 ± 0.015^q–t^	0.16 ± 0.01	1.01 ± 0.06	1.62 ± 0.03^n^
B2	16.75 ± 0.03^h^	0.14 ± 0.010^tu^	0.16 ± 0.01	1.08 ± 0.05	1.60 ± 0.02^no^
B3	16.76 ± 0.04^h^	0.18 ± 0.010^o–r^	0.19 ± 0.01	0.98 ± 0.03	1.63 ± 0.02^m^
B4	16.81 ± 0.05^h^	0.20 ± 0.010^m–o^	0.20 ± 0.01	1.00 ± 0.06	1.57 ± 0.01^op^
B5	16.76 ± 0.03^h^	0.15 ± 0.006^r–u^	0.21 ± 0.02	0.99 ± 0.04	1.57 ± 0.02^p^
E1	10.86 ± 0.02^i^	0.38 ± 0.012^i^	Trace	1.22 ± 0.07	1.31 ± 0.02^t^
E2	10.83 ± 0.04^ij^	0.45 ± 0.015^f^	Trace	1.17 ± 0.05	1.35 ± 0.02^t^
E3	10.82 ± 0.06^ij^	0.41 ± 0.015^gh^	Trace	1.19 ± 0.04	1.32 ± 0.01^t^
E4	10.77 ± 0.02^j^	0.39 ± 0.021^hi^	Trace	1.20 ± 0.05	1.27 ± 0.02^u^
E5	10.84 ± 0.02^ij^	0.43 ± 0.015^g^	Trace	1.18 ± 0.08	1.27 ± 0.02^u^
M1	22.74 ± 0.02^de^	0.02 ± 0.001^v^	Trace	1.05 ± 0.03	1.82 ± 0.02^k^
M2	22.77 ± 0.06^cd^	0.02 ± 0.002^v^	Trace	1.02 ± 0.03	1.78 ± 0.01^kl^
M3	22.70 ± 0.04^e^	0.02 ± 0.001^v^	Trace	1.02 ± 0.02	1.77 ± 0.02^lm^
M4	22.77 ± 0.03^cd^	0.02 ± 0.001^v^	Trace	1.00 ± 0.02	1.84 ± 0.02^j^
M5	22.82 ± 0.04^c^	0.02 ± 0.002^v^	Trace	1.06 ± 0.05	1.81 ± 0.02^k^
I1	23.09 ± 0.03^ab^	0.66 ± 0.020^c–e^	Trace	1.23 ± 0.07	3.10 ± 0.02^de^
I2	23.08 ± 0.03^ab^	0.64 ± 0.020^e^	Trace	1.24 ± 0.04	3.12 ± 0.02^de^
I3	23.09 ± 0.04^ab^	0.68 ± .015^b–d^	Trace	1.18 ± 0.05	3.13 ± 0.02^d^
I4	23.06 ± 0.02^b^	0.70 ± 0.031^ab^	Trace	1.19 ± 0.06	3.08 ± 0.02^e^
I5	23.13 ± 0.02^a^	0.65 ± 0.010^de^	Trace	1.17 ± 0.07	3.09 ± 0.02^e^
BO1	6.34 ± 0.03^l^	0.14 ± 0.010^s–u^	Trace	1.76 ± 0.09	3.21 ± 0.03^ab^
BO2	6.38 ± 0.03^l^	0.15 ± 0.010^s–u^	Trace	1.72 ± 0.05	3.19 ± 0.02^bc^
BO3	6.38 ± 0.03^l^	0.13 ± 0.006^u^	Trace	1.69 ± 0.04	3.19 ± 0.02^bc^
BO4	6.34 ± 0.04^l^	0.17 ± 0.010^p–s^	Trace	1.75 ± 0.08	3.17 ± 0.02^c^
BO5	6.27 ± 0.02^m^	0.18 ± 0.006^o–q^	Trace	1.73 ± 0.07	3.24 ± 0.02^a^

The leaves of cultivated licorice genotypes contained rutin ranging from 1.27 ± 0.02 mg/g DW in the E4 genotype to 3.24 ± 0.02 mg/g DW in the BO5 genotype (Table 4). Comparison between rutin values in wild populations and cultivated genotypes indicated a decrease in rutin content in all cultivated genotypes except for the genotypes of Ilam and Bojnourd populations.

### Total phenol and total flavonoid content, and antioxidant activity

Considerable variation was found among licorice genotypes for TPC, TFC, and antioxidant activity (IC_50_) (Table 5). Total phenol content and TFC varied from 4.20 ± 0.10 to 11.80 ± 0.60 and 2.10 ± 0.20 to 7.67 ± 0.21, respectively. The greatest TPC and TFC were observed in the genotype K2 of the Kashmar population. The lowest TPC and TFC belonged to the genotypes of the Marvest population, specifically to the genotype M5. The maximum and minimum antioxidant activity was observed in the genotypes of the Kashmar and Marvest populations, respectively. The results of the ferric reducing antioxidant power was also confirmed the result of DPPH assay, wherein the genotypes (K1-K5) of the Kashmar population showed outstanding antioxidant activity (Table 5).

**Table 5:** Total phenol content (TPC), total flavonoid content (TFC), and antioxidant activity of the cultivated Iranian *Glycyrrhiza glabra* genotypes.

Genotype code	TPC (mg GAE/g DW)	TFC (mg RE/g DW)	IC_50_ (μg/ml)	FRAP (µmol Fe^2+^/g DW)
K1	10.73 ± 0.65	7.17 ± 0.12	40.90 ± 0.20	151.42 ± 8.5
K2	11.80 ± 0.60	7.67 ± 0.21	38.40 ± 0.20	149.25 ± 7.8
K3	11.13 ± 0.85	6.73 ± 0.21	44.97 ± 0.21	146.88 ± 7.7
K4	11.17 ± 0.76	6.80 ± 0.10	42.70 ± 0.20	152.85 ± 8.9
K5	11.30 ± 0.36	7.33 ± 0.21	39.27 ± 0.80	150.12 ± 8.2
TK1	7.63 ± 0.15	4.33 ± 0.21	74.73 ± 0.21	89.45 ± 4.3
TK2	7.23 ± 0.06	4.23 ± 0.12	76.50 ± 0.30	92.54 ± 4.5
TK3	7.80 ± 0.10	5.03 ± 0.12	73.37 ± 0.40	98.12 ± 5.2
TK4	7.47 ± 0.12	4.40 ± 0.30	74.47 ± 0.15	97.15 ± 5.1
TK5	8.03 ± 0.15	4.27 ± 0.31	75.23 ± 0.35	88.65 ± 4.2
TF1	6.67 ± 0.12	5.17 ± 0.21	76.20 ± 0.10	89.36 ± 4.3
TF2	6.73 ± 0.21	4.23 ± 0.25	78.87 ± 0.06	86.25 ± 4.5
TF3	6.13 ± 0.06	4.20 ± 0.17	80.27 ± 0.15	84.74 ± 4.4
TF4	6.37 ± 0.12	4.37 ± 0.12	77.80 ± 0.10	86.26 ± 4.6
TF5	6.53 ± 0.40	3.90 ± 0.20	82.23 ± 0.15	77.98 ± 3.9
B1	6.37 ± 0.25	3.63 ± 0.21	90.20 ± 0.10	75.11 ± 3.6
B2	5.70 ± 0.26	3.27 ± 0.06	93.20 ± 0.26	71.23 ± 3.1
B3	6.90 ± 0.10	3.67 ± 0.25	89.17 ± 0.12	70.25 ± 3.2
B4	5.40 ± 0.20	3.73 ± 0.12	88.40 ± 0.30	86.26 ± 4.3
B5	5.30 ± 0.20	3.53 ± 0.15	91.23 ± 0.15	78.35 ± 4.1
E1	8.50 ± 0.60	4.27 ± 0.21	72.20 ± 0.10	100.15 ± 5.3
E2	8.20 ± 0.20	5.17 ± 0.12	66.47 ± 0.40	99.25 ± 4.8
E3	8.63 ± 0.23	4.63 ± 0.21	69.77 ± 0.31	97.24 ± 4.9
E4	9.20 ± 0.10	4.43 ± 0.21	70.17 ± 0.12	101.85 ± 4.8
E5	7.87 ± 0.29	4.67 ± 0.21	68.60 ± 0.26	103.41 ± 5.1
M1	4.90 ± 0.20	2.23 ± 0.15	99.97 ± 0.12	70.45 ± 3.3
M2	5.10 ± 0.10	2.40 ± 0.30	98.60 ± 0.26	68.25 ± 3.1
M3	4.60 ± 0.44	2.27 ± 0.12	101.60 ± 0.10	66.78 ± 3.0
M4	4.37 ± 0.15	2.13 ± 0.21	100.23 ± 0.35	68.88 ± 3.2
M5	4.20 ± 0.10	2.10 ± 0.20	99.73 ± 1.00	69.45 ± 3.3
I1	10.00 ± 0.20	5.53 ± 0.15	57.77 ± 0.15	116.25 ± 6.2
I1	9.60 ± 0.36	5.00 ± 0.10	60.37 ± 0.70	116.64 ± 5.8
I1	9.10 ± 0.36	6.00 ± 0.26	55.60 ± 0.36	118.19 ± 6.3
I1	8.87 ± 0.21	6.57 ± 0.21	52.47 ± 0.12	121.22 ± 6.1
I1	9.20 ± 0.30	5.33 ± 0.21	59.20 ± 0.10	115.23 ± 5.6
BO1	6.27 ± 0.15	3.17 ± 0.12	95.43 ± 0.32	73.25 ± 3.9
BO2	5.67 ± 0.23	3.17 ± 0.21	95.17 ± 0.06	74.11 ± 4.3
BO3	7.20 ± 0.10	2.80 ± 0.10	96.27 ± 0.38	72.46 ± 4.1
BO4	5.77 ± 0.15	3.23 ± 0.15	94.40 ± 0.30	73.15 ± 4.2
BO5	5.10 ± 0.10	3.53 ± 0.21	93.40 ± 0.17	75.55 ± 5.1

### Cluster analysis and correlation of traits

**Figure 4:**
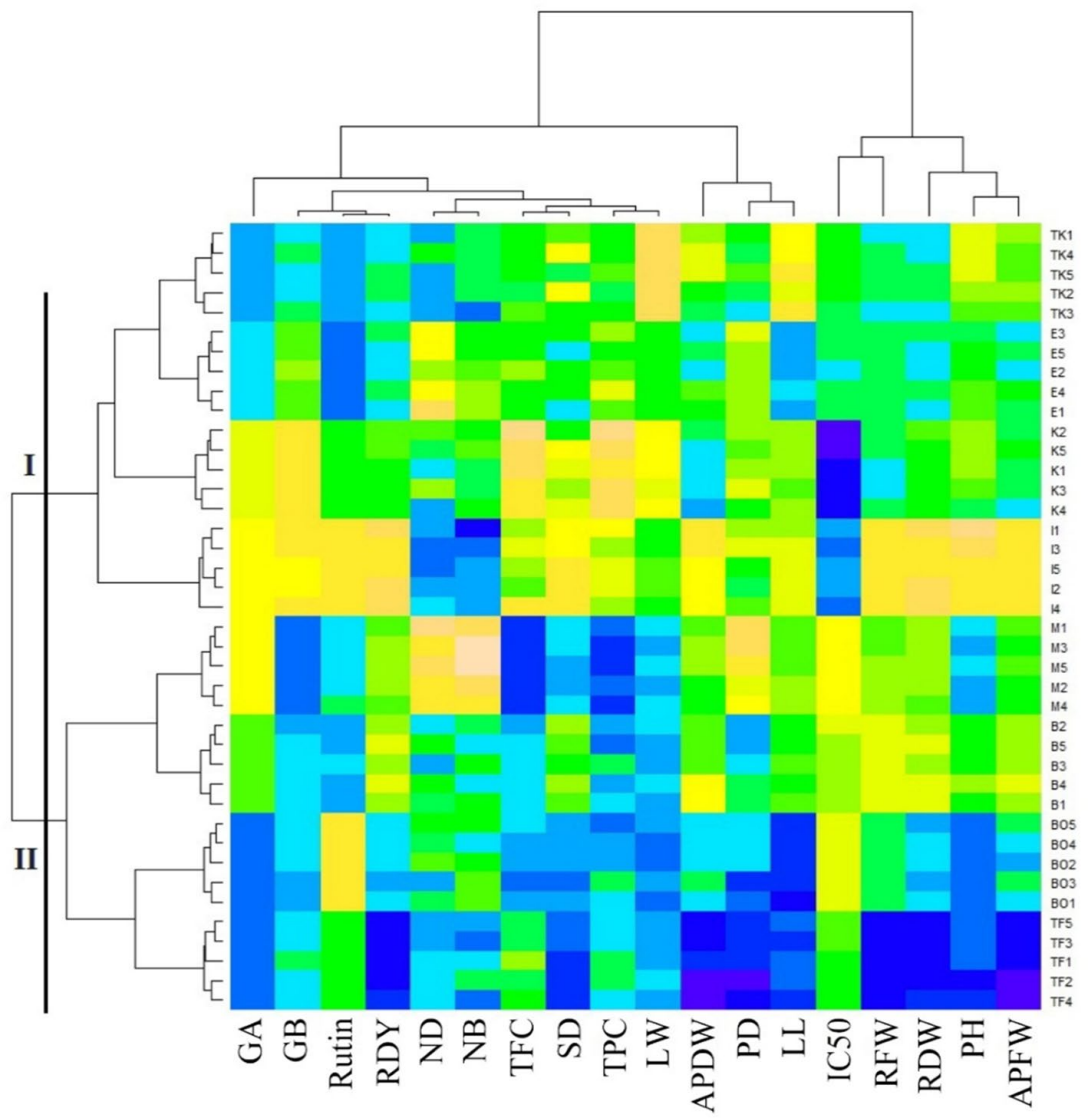
The heatmap cluster analysis of cultivated licorice (*Glycyrrhiza glabra* L*.*) genotypes based on all phytochemical and morphological traits. *GA* glycyrrhizic acid content, *GB* glabridin content, *Rutin* rutin content, *RDY* root dry yield, *ND* no. of node, *NB* no. of branches, *TFC* total flavonoid content, *SD* stem diameter, *TPC* total phenol content, *LW* leaf width, *APDW* aerial part dry weight, *PD* plant diameter, *LL* leaf length, *IC*_*50*_ half maximal inhibitory concentration, *RFW* root fresh weight, *RDW* root dry weight, *PH* plant height, *APFW* aerial part fresh weight.

The heatmap cluster analysis based on morphological and phytochemical traits clustered all forty genotypes into two major categories. The cluster I divided into two subgroups, wherein the genotypes of Takestan, Eghlid, and Kashmar populations were more similar to each other and placed in one subgroup. The genotypes of Ilam formed exclusively the other subgroup in cluster I. By looking at the measured morphological traits, it was found that genotypes from Takestan, Eghlid, and Kashmar populations dedicated similar values and thus grouped into the first subgroup of cluster I. Cluster II included genotypes from Marvest, Bajgah, Bojnourd, and Taft populations, which showed approximately homological value in most morphological traits (Fig. 4).

**Figure 5:**
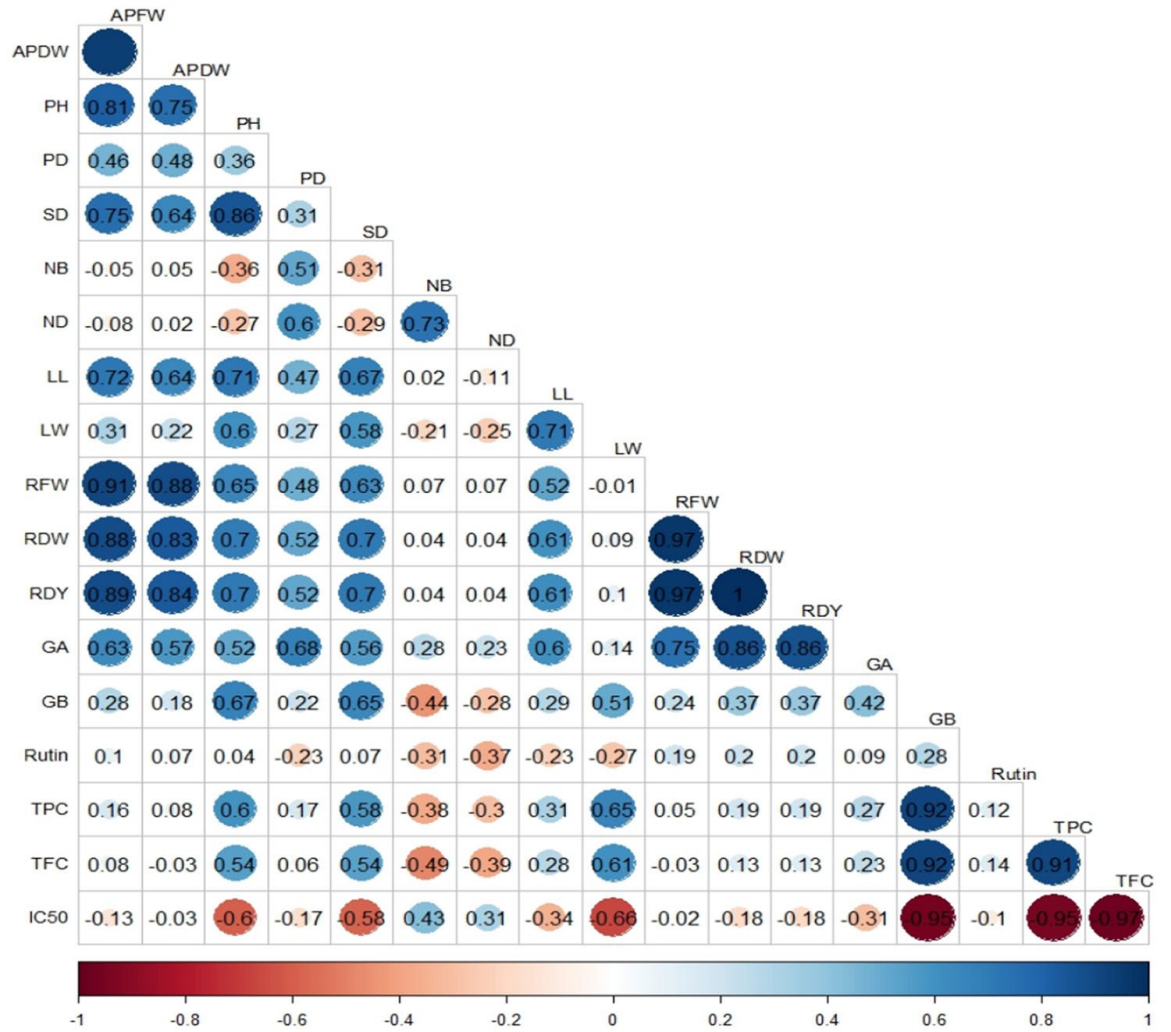
The Corrplot schema based on simple correlation analysis between studied traits. *APDW* aerial part dry weight, *PH* plant height, *PD* plant diameter, *SD* stem diameter, *NB* no. of branches, *ND* no. of node, *LL* leaf length, *LW* leaf width, *RFW* root fresh weight, *RDW* root dry weight, *RDY* root dry yield, *GA* glycyrrhizic acid content, *GB* glabridin content, *Rutin* rutin content, *TPC* total phenol content, *TFC* total flavonoid content, *IC*_*50*_ half maximal inhibitory concentration.

Conducting the simple correlation analysis between the studied traits indicated that GA content was correlated with plant diameter (*r* = 0.68), root fresh weight (*r* = 0.75), and root dry weight and yield (*r* = 0.86). Glabridin content was positively associated with the plant height (*r* = 0.67), and stem diameter (*r* = 0.65) traits. A significant negative correlation was observed between the glabridin content and IC_50_ (*r* = − 0.95). IC_50_ was also negatively associated with leaf width (*r* = − 0.66), TPC (*r* = − 0.95), and TFC (*r* = − 0.97) (Fig. 5).

**Figure 6:**
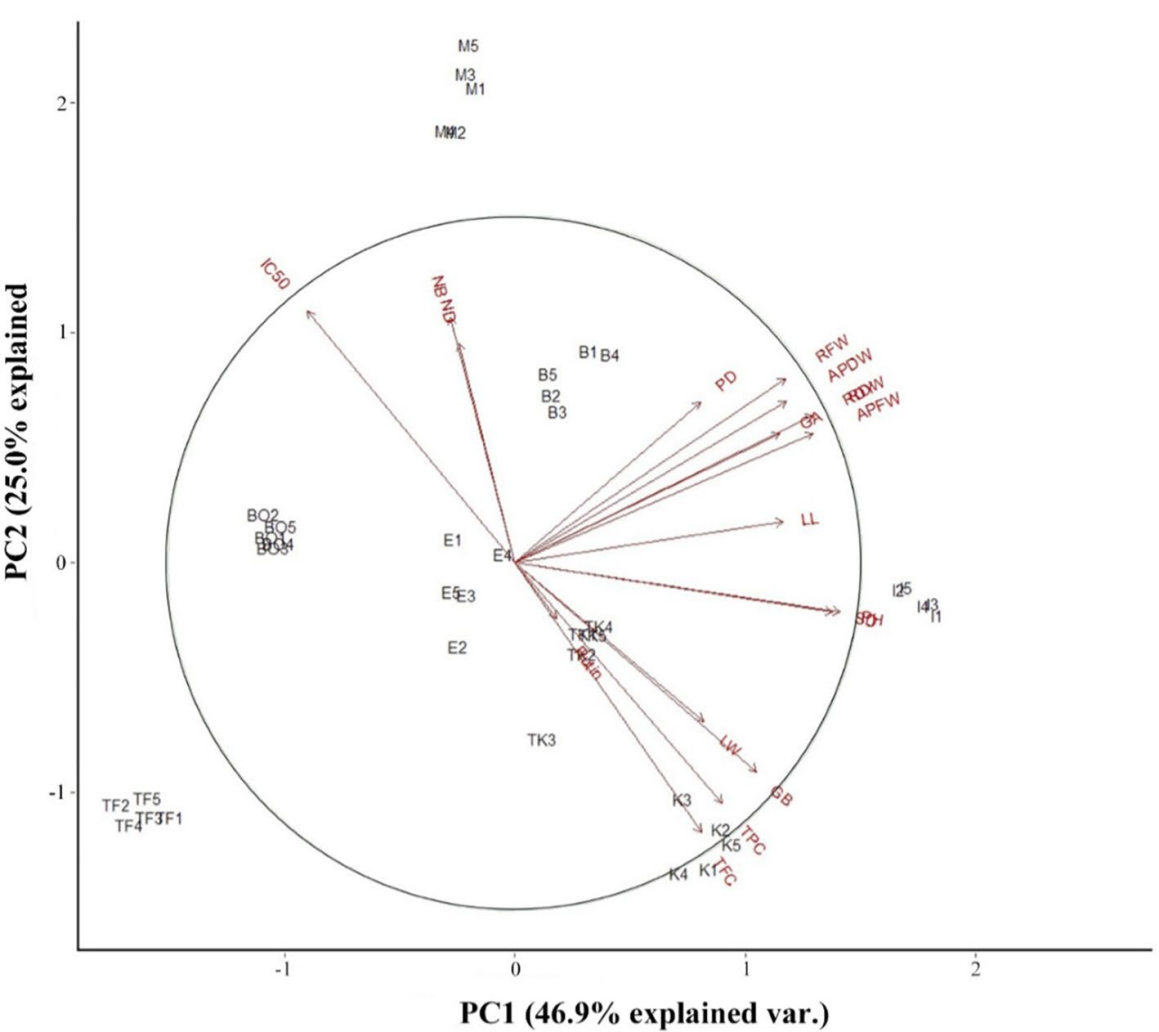
Biplot of PCA based on all measured phytochemical and morphological traits.

Eighteen phytochemical and phenotypic attributes of *G. glabra* were defined in three major components with expounding 84.0% of the total variance based on principal component analysis (PCA). The first component (PC1), 46.9% of the total variance, displays some morphological traits such as plant height and stem diameter had the most portion of the variance. In the PC_2_, explaining 25.0% of the total variance, antioxidant parameters such as TPC, TFC, and antioxidant activity (IC_50_) had the maximum portion of variance (Table 6). Biplot analysis concerning both PC1 and PC2 also divided the genotypes into eight distinct groups based on their habitats (Fig. 6).

**Table 6:** Principal component analysis (PCA) for the studied traits of Iranian *Glycyrrhiza glabra* genotypes.

Variable	Component		
	1	2	3
Aerial part fresh weight	0.295	− 0.176	0.151
Aerial part dry weight	0.268	− 0.219	0.138
Plant height	0.322	0.067	0.046
Plant diameter	0.183	− 0.218	− 0.406
Stem diameter	0.315	0.067	0.050
No. of branches	− 0.064	− 0.332	0.374
No. of node	− 0.056	− 0.297	− 0.417
Leaf length	0.265	− 0.056	− 0.148
Leaf width	0.187	0.215	− 0.294
Root fresh weight	0.268	− 0.250	0.192
Root dry weight	0.295	− 0.201	0.136
Root dry yield	0.295	− 0.200	0.135
Glycyrrhizic acid content	0.262	− 0.175	− 0.114
Glabridin content	0.239	0.284	− 0.063
Rutin content	0.041	0.075	0.444
Total phenol content	0.205	0.326	− 0.171
Total flavonoid content	0.184	0.366	− 0.128
Antioxidant activity (IC_50_)	− 0.206	− 0.342	0.181
Eigenvalue	8.435	4.497	2.190
% of variance	46.9	25.0	12.2
Cumulative %	46.9	71.8	84.0

## Discussion

Reasons such as the impact of different environmental conditions on SMs and the increasing limitations of harvesting from nature as well as the necessity for sustainable and uniform production of SMs have made it necessary to introduce wild MAPs into cultivation systems. In the present study, to homogenize the edaphic and environmental conditions, different wild-collected licorice genotypes were first cultivated in the same location and then investigated for the desired phytochemical and morphological traits. Some reports have indicated the use of selective breeding to introduce superior varieties for *Ephedra sinica*^32^ and *Panax ginseng*^33^.

The plant genotypes from Ilam, Bajgah, Eghlid, and Kashmar populations exhibited admissible vegetative growth, in contrast to genotypes from the Taft population which did not exhibit good vegetative growth and yield properties. The cultivation site had a lower average annual temperature than the natural habitat of the genotypes collected from Taft, which could explain the reduction in yield and vegetative growth in the new environment. The minimum coefficient of diversity for morphological traits was recorded for plant height. Sharifi et al.^34^ reported that the least variability in natural licorice populations was related to the legume width trait. The homogeneity of cultivated wild populations in Shiraz with respect to stem diameter has been previously reported^28^.

The yield and metabolite production of populations in such a homogeneous environment can be considered their genetic manifestation because of the elimination of different environmental effects. Estimating the diversity of landraces and wild populations of MAPs species is the most important step in the selecting of the top genotypes for introducing them to the cultivation system and achieving successful breeding programs. Owing to the high diversity of these genetic resources, classical selective breeding methods are still the most common method of MAPs breeding and development of cultivation and production. Different strains of *G. uralensis* were subjected to selective breeding, in which A-19 and G-6 were commercially released with a high GA content and strong growth, respectively^35^. In Japan, using the selective method superior clones with high GA content have been introduced from *G. uralensis*^36^.

In the present study, a significant decrease in GA and glabridin content was observed in the cultivated licorice genotypes from eight populations compared with its wild-grown populations. The previous studies on Chinese licorice (*G. uralensis*) have proven that the root quality and effective substances of the cultivated plant was greatly reduced compared with the wild-grown plants, wherein the content of main flavonoids was much higher in rhizomes of wild plants^23,37^. This fluctuation in the metabolites content can be due to the difference in the growth environment of plants, as plants in their natural habitat are faced with various types of stress and different factors. These different biotic and abiotic factors stimulate the plant to produce more metabolites. It has also been reported that the roots of wild licorice plants that did not receive artificial fertilizer and other inputs expanded well and produced strong and thick roots^23^, which can be a reason for the increase of metabolites in wild types compared with cultivated ones. The decrease in the quality of cultivated licorice indicates that the breeding programs to increase the main metabolites of this plant should be pursued more urgently. It has been reported that the increase of licorice root GA and glabridin has a direct relationship with the age and diameter of the rhizome^38^. The average content of glabridin in the cultivated Iranian licorice genotypes was about 0.03%, while its average content in licorice root has been previously reported to be 0.2%^39^. Phytochemical components in the underground parts of licorice also vary based on variety, species, origin, and growing conditions^40,41^. Zhang et al.^42^ considered edaphic properties to be more important than other factors affecting the production of metabolites in licorice rhizomes. Statti et al.^43^ have also stated that the heterogeneity of solar exposure, altitude, and latitude could be the reason for the observed phytochemicals variation in nine collected populations from Italy.

Variation in the content of GA (3.3–6.1% DW) and glabridin (0.08–0.35% DW) has been reported in different licorice populations from Uzbekistan^44^. In the present study, GA and glabridin varied from 5.00 to 23.13 mg/g DW and from 0.02 to 0.72 mg/g DW among cultivated genotypes, respectively.

The standard and stable production of GA and glabridin through a variety improvement, in addition to high economic profit, can meet the needs of consumer industries. This can also eliminate the country's need to import enriched GA and glabridin extracts from abroad. Most studies on licorice have focused on GA as the most well-known metabolite of the plant and some varieties were introduced with high GA in other countries. Although glabridin has been considered a valuable metabolite, some nodules need to be untied before further use. For example, the pharmacological effects of glabridin have often been proven in clinical trials on cells and animals, and therefore further research on human volunteers or participants is needed. Also, not much information is available on the toxicity of glabridin^13^. On the other hand, due to the low content of glabridin in the plant, the superior and suitable genotype can be further exploited in novel agronomical systems like aeroponic, hydroponic, vertical farming, and biotechnological methods like plant hairy root culture for its efficient production.

Except for genotypes from Ilam and Bojnourd populations, the rutin content in the leaves of cultivated licorice genotypes decreased compared with those in the natural habitat. Dražić et al.^45^ have claimed that the altitude of the growth location of buckwheat (*Fagopyrum esculentum* Moench) affected the plant rutin content. Cultivation of the studied licorice genotypes at the cultivation site with lower altitudes than their natural habitat can justify the reduction in rutin production and accumulation in the plant leaves. Two main categories of flavonoids including isoquercitrin and rutin classes were reported by Hayashi et al.^44^ in the aerial parts of licorice. The present results also revealed that the studied Iranian licorice populations are classified as a rutin-type class.

In the present study, considerable variation in terms of antioxidant parameters was observed among genotypes. Total phenol content, TFC, and antioxidant activity of the plant have been previously reported from other parts of the world^46–48^. Total phenol content (TPC) (13.23–37.27 mg GAE/g DW) and TFC (2.69–5.90 mg QE/g DW) of licorice from Serbia has also been reported^43^. In a previous study, the TFC of licorice root extracts (91.75 ± 6.61 mg Catechin equivalent/g) was reported for its proper antioxidant activity^49^. The finding of the present study showed that the genotypes (K1–K5) from the Kashmar population with the highest TPC and TFC donated the greatest antioxidant activity which is in agreement with previous findings^50–53^. This trend was also observed between the studied genotypes. Outstanding antioxidant activity was also found in genotypes (K and I) with high glabridin content. It should be noted that the high antioxidant power of glabridin has already been proven^13,14,54,55^.

In this study, the all forty genotypes placed into two major categories by the cluster analysis. In the cluster analysis of wild populations of this plant from Iran, the populations of western and northwestern regions were placed in cluster one and the populations of central regions were placed in cluster two^28^. On the contrary, in another study, the cluster analysis of wild licorice individuals based morphological and phytochemical traits showed that the genotypes were not grouped according to their geographical regions^56^. With study of the correlation between environmental factors and bioactive compounds of *G. uralensis*, it has been determined that increasing the duration of sunlight caused an increase in glycyrrhizin, while decreasing rainfall led to the accumulation of liquiritigenin and isoliquiritigenin^57^. In the study of the morphological characteristics of different species of the genus *Glycyrrhiza*, the distinction of *G. glabra* from other species was determined using cluster analysis^58^. In the study of wild licorice populations collected from different parts of Kazakhstan, these populations were classified into three clusters in terms of morphological characteristics, wherein the first and second clusters were related to *G. uralensis* and *G. glabra*, respectively, while the third group was intermediate^59^. Eghlima et al.^56^ reported that there was a significant direct relationship between root and shoot dry weight, while no correlation was observed among leaf characteristics. In the previous study, a negative correlation between glabridin content and the number of nodes was reported^28^. The significant positive correlation between glabridin content and antioxidant activity observed in this study was consistent with previous studies, which confirmed the potential use of glabridin as a natural antioxidant^14,60^.

## Conclusion

In this study, by cultivating the licorice genotypes in the same environment and uniform geographic and soil conditions, the content of their effective compounds was compared with the wild genotypes of the plant in Iran. The results indicated that a significant decrease in GA and glabridin content was observed in the cultivated licorice genotypes compared with its wild-grown genotypes. The average content of GA and glabridin in the cultivated Iranian licorice genotypes was recorded about 14.35 and 0.32 mg/g DW, respectively, while their average content in the roots of the wild licorice genotypes has been previously reported to be 30.16 and 3.06 mg/g DW. The present study showed that the genotype I5 from the Ilam population was rich in GA content. The glabridin content in the K2 genotype was higher than in other genotypes but did not show significant differences with the I4 genotype. According to the obtained root yield among the studied genotypes, the genotype I4 (3.07 t/ha) belonging to the Ilam population can be introduced as a glabridin-rich genotype. Therefore, I5 and I4 genotypes can be candidates for further use in cultivation, breeding, and biotechnological programs.

### Supplementary Information

Supplementary Tables.

## Data Availability

The dataset generated during and/or analyzed during the current study are available from the corresponding author on reasonable request.
