# Supplementary material for: Introducing the glycyrrhizic acid and glabridin rich genotypes from the cultivated Iranian licorice (Glycyrrhiza glabra L.) populations to exploit in production systems
Source: Sci Rep. 2024 May 14;14:11034. doi: 10.1038/s41598-024-61711-1 (PMC11094143; doi:10.1038/s41598-024-61711-1)
Supplement: Supplementary file 1 — Supplementary Tables. [file 41598_2024_61711_MOESM1_ESM.docx]

| Table S1. The analysis of variance (ANOVA) for yield and morphological traits of cultivated licorice genotypes | | | | | | | | | | | | | |
| --- | --- | --- | --- | --- | --- | --- | --- | --- | --- | --- | --- | --- | --- |
| Source | DF | Mean Square | | | | | | | | | | | |
|  |  | Dependent Variables | | | | | | | | | | | |
|  |  | APFW | APDW | PH | PD | SD | NB | ND | LL | LW | RFW | RDW | RDY |
| Treatment | 39 | 320.53^**^ | 108.68^**^ | 252.07^**^ | 48.95^**^ | 2.87^**^ | 23.58^**^ | 16.91^**^ | 90.28^**^ | 21.14^**^ | 511.49^**^ | 152.29^**^ | 0.34^**^ |
| Block | 2 | 2.78 | 3.06 | 7.44 | 0.81 | 0.17 | 1.10 | 0.16 | 0.36 | 0.03 | 0.27 | 0.38 | 0.00 |
| Error | 78 | 1.35 | 0.92 | 2.85 | 1.29 | 0.06 | 0.89 | 0.86 | 0.38 | 0.05 | 0.35 | 0.20 | 0.00 |

^**^ Significant at α-level = 0.1; APFW: Aerial part fresh weight, APDW: Aerial part dry weight, PH: Plant height, PD: Plant diameter, SD: Stem diameter, NB: No. of branches, ND: No. of node, LL: Leaf length, LW: Leaf width, RFW: Root fresh weight, RDW: Root dry weight, RDY: Root dry yield.

| Table S2. The analysis of variance (ANOVA) for phytochemical traits of cultivated licorice genotypes | | | | | | | |
| --- | --- | --- | --- | --- | --- | --- | --- |
| Source | DF | Mean Square | | | | | |
|  |  | Dependent Variables | | | | | |
|  |  | GA | GB | Rutin | TPC | TFC | IC_50_ |
| Treatment | 39 | 154.562^**^ | 0.167^**^ | 1.367^**^ | 13.008^**^ | 6.537^**^ | 1073.399^**^ |
| Block | 2 | 0.002 | 6.166E-5 | 0.000 | 0.235 | 0.020 | 0.013 |
| Error | 78 | 0.001 | 0.000 | 0.000 | 0.100 | 0.037 | 0.106 |

^**^ Significant at α-level = 0.1; GA: Glycyrrhizic acid content, GB: Glabridin content, Rutin: Rutin content, TPC: Total phenol content,

TFC: Total flavonoid content, IC_50_: Half maximal inhibitory concentration
